# The Role of Copper Homeostasis at the Host-Pathogen Axis: From Bacteria to Fungi

**DOI:** 10.3390/ijms20010175

**Published:** 2019-01-05

**Authors:** Chao Li, Yanjian Li, Chen Ding

**Affiliations:** College of Life and Health Sciences, Northeastern University, Shenyang 110000, China; fMssop_li@163.com (C.L.); leeyanjian@163.com (Y.L.)

**Keywords:** copper, fungal pathogen, bacterial pathogen, immunity

## Abstract

Copper is an essential trace element participating in many vital biological processes, however it becomes a toxic agent when in excess. Thus, precise and tight regulation of copper homeostasis processes, including transport, delivery, storage, detoxification, and efflux machineries, is important, ensuring that only the amount needed to sustain basic biological functions and simultaneously prevent copper toxicity in the cell is maintained. Numerous exciting studies have revealed that copper plays an indispensable role at the microbial pathogen-host axis for entities ranging from pathogenic bacteria to deadly fungal species. Analyses of copper homeostases in bacteria and fungi extensively demonstrate that copper is utilized by the host immune system as an anti-microbial agent. The expression of copper efflux and detoxification from microbial pathogens is induced to counteract the host’s copper bombardment, which in turn disrupts these machineries, resulting in the attenuation of microbial survival in host tissue. We hereby review the latest work in copper homeostases in pathogenic bacteria and fungi and focus on the maintenance of a copper balance at the pathogen-host interaction axis.

## 1. Copper and Life

Copper is an essential trace metal found in almost all forms of life, from bacteria to mammals. A computational algorithm analysis estimates that ~0.3% of bacterial proteomes are potential copper-binding proteins (cuproproteins), and the average adult male human body contains about 100 mg of copper [1,2,3]. The ionic conversion between Cu^2+^ and Cu^+^ provides a rich and potent redox reaction, acting as a reactive center for many critical enzymatic catalytic reactions that are important for numerous vital biological processes such as melanin formation, respiration, iron uptake, iron transportation, and superoxide detoxification [4,5]. The range of copper-targeting processes is broad, rendering this element critical for fruit ripening, embryonic development, neuron function, heart development, and immunity. The ethylene sensing receptor (ETR1) in plants uses copper as a cofactor for ethylene binding and signaling [6]. A deficiency leads to embryonic death and severe cardiomyopathy [7,8]. Neuron activation is associated with the redistribution of intracellular copper [9]. Host immune cells such as macrophages use copper as an anti-invading microbial agent [10]. Additionally, copper is used as a structural cofactor that drives conformational changes in transcription factors to differentially modulate gene expression [11].

Despite the indispensable role of copper in cellular functioning, a dysregulation or imbalance in its metabolism often results in severe and irreversible cellular damage, leading to diseases in mammals. Important proteins that are involved in the formation of neurodegenerative diseases include cuproproteins such as the amyloid precursor protein (APP) and prion protein (PrP) [12,13]. The APP participates in neurological development, and its abnormal cleavage generates the Aβ_1–42_ peptide that aggregates in senile plaques, a causative agent in Alzheimer’s disease. Evidence suggests that copper is involved in the formation of Aβ aggregation, and copper chelator therapy has been proposed for clinical treatment. The prion protein binds copper via the N-terminal octapeptide repeats, yet its function remains unclear. Furthermore, mutations in important copper transporting systems lead to the formation of Menkes and Wilson’s diseases, and many types of cancers are related to alterations in the intracellular copper content [4,13].

## 2. Copper and Immunity

Both clinical investigations and molecular validations have pointed to the critical nature of copper in modulating host immunity. Generally, its contribution to immunity is through two avenues: (1) Participating in the development and differentiation of immune cells and (2) providing anti-fungal properties via either metal sequestration machineries in the host or as bombardment. A study of copper metabolism in cattle revealed that reducing dietary copper impairs the ability of neutrophils to kill *Candida albicans* [14,15]. A subsequent study in rats found similar results: Respiratory burst and anti-*Candida* activity were significantly impaired after 1 week on a copper-deficient diet. A recent clinical case study demonstrated that after consuming a copper supplement for 2 months, a patient with neutropenia drastically recovered neutrophil counts, indicating that copper deficiency was a cause of this case of neutropenia and that copper is involved in monocyte progenitor cell differentiation [16].

Studies of mycobacterium infection using X-ray microprobe analyses demonstrated that copper is concentrated in the phagosomal compartment, providing anti-bacterial bombardment [10]. Indeed, interferon-gamma activated macrophage-like cells extensively induce protein expression of mammalian copper transporters. In turn, *Escherichia coli* strains lacking functional CopA copper-transporting ATPase are extremely susceptible to macrophage killing [17]. In vivo analysis using a candidiasis kidney infection model revealed that tissue copper levels increase at the initial stage of infection, then decline [18]. Pulmonary infection and meningitis analyses in *Cryptococcus neoformans* demonstrated that copper transporters showed reciprocal roles in host tissues: Distinct regulation mechanisms for copper homeostasis in hosts were found to counteract fungal invasion [19]. In this review, we focus on homeostasis in pathogenic bacterial and fungal cells, and we elucidate their functions at the host-pathogen axis.

## 3. Copper Homeostasis in Pathogenic Bacteria

**Figure 1:**
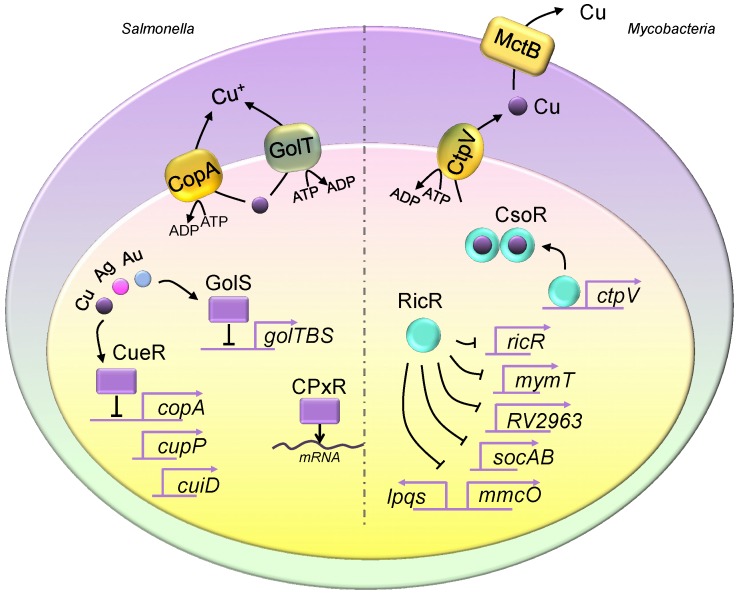
Copper homeostasis in *Salmonella* and *Mycobacteria*. The *Salmonella* genome encodes two copper exporter genes, and they produce two P-type ATPase copper efflux transporters, CopA and GolT. Both copper exporters pump copper from the cytosol into the periplasmic space. The expression of *copA* is regulated by CueR, which is a transcriptional repressor, and it is activated upon copper binding. CueR also controls the expression of *cueP* and *cuiD*. The translation of *cueP* is controlled by CpxR, which recruits RNA polymerase. The expression of the *golTBS* regulon is controlled by GolS. Similar to CueR, GolS responds to elevated copper levels. Additionally, both GolS and CueR sense other metals such as gold and silver. *Mycobacteria* utilize CtpV and mycobacterial copper transporter protein B (MctB) to eliminate intracellular copper, where CtpV pumps copper from the cytosol into the periplasmic space, and MctB pumps copper extracellularly. The two copper regulatory mechanisms are the CsoR and RicR systems. Similar to those seen in *Salmonella*, CsoR and RicR are transcriptional repressors, and dissociate from promoters of copper regulon genes when bound to copper. The copper-bound CsoR forms a homodimer. The apo form of CsoR binds to the regulatory promoter sequences.

Studies of bacteria greatly facilitate and accelerate discovery of the roles of copper at the infection axis. Using *mycobacterium*, *E. Coli*, and *salmonella*, it is now known that macrophages modulate the expression of copper regulons and mobilize copper to create a copper overdose environment for invading bacteria [10,17]. On the other hand, bacteria cells have evolved and now provide complex and efficient regulation mechanisms to neutralize toxic copper. From a large number of bacteria species, we review copper homeostasis and its regulation in two major human pathogenic bacteria, *Salmonella enterica sv.* Typhimurium (Stm), and *Mycobacterium tuberculosis*, and we will focus on the bactericidal activities of copper in the host (Figure 1).

### 3.1. The Copper Transport System in Pathogenic Bacteria

Although a copper importing system has been reported in many eukaryotic organisms such as those comprising the Ctr family, the bacterial copper influx machinery is poorly understood, primarily because bacterial cuproproteins tend to localize in the periplasmic and extracellular spaces [5,20]. In fact, the bacterial copper efflux system plays a predominant role in regulating pathogen fitness during infection. The addition of a specific copper chelate molecule acid, enhances the intracellular survival of *Salmonella* in macrophages [21]. Conditionally deleting a mammalian P-type ATPase, ATP7A, results in reduced phagosomal copper content and a dampening of the expression of *Salmonella* copper efflux encoding genes [22]. These works have extensively demonstrated that the copper efflux components are critical bacterial virulence determinants.

The bacterial copper efflux system was extensively demonstrated in *Enterococcus hirae*, and it was further applied to other bacteria. CopB and CopA are mammalian ATP7A-like P-type ATPases, similar to the fungal Golgi copper transporter Ccc2 [23,24,25,26,27]. However, the Cu-binding motifs of CopB and CopA share low similarity, where a histidine-rich N-terminal domain participates in Cu binding of CopB and CopA possess a Cys-Pro-Cys Cu-binding motif [28,29]. Their functions are to pump intracellular copper into the extracellular space. In pathogenic *Salmonella* species, mediation of the copper efflux is achieved through CopA and GolT, where GolT is also a P-type ATPase [30] (Figure 1). Both efflux transporters work in a functionally redundant manner, and disruption of gene expression results in hypersensitivity to copper treatment and reduced bacteria survival in macrophage-like cells (RAW264.7). Macrophages employ ATP7A to concentrate copper levels in the phagosomal compartment. The expressions of both *copA* and *golT* are dramatically induced in primary macrophages. Further analysis indicates that the induced gene expression is abolished in *ATP7A^LysMcre^* mouse macrophages, showing impairment of copper transport into the phagosomal compartment [22].

CtpV is a cytoplasmic membrane-bound Cu^+^ efflux transporter in *Mycobacteria* [5,20] (Figure 1). Research has demonstrated that the gene expression of *ctpV* is in response to elevated copper levels, and disruption of *ctpV* triggers a copper-sensitive phenotype and reduces bacterial growth in guinea pig lung tissues in the early stage of infection [31]. Furthermore, animal survival assays showed that animals infected with the *ctpV* deletion strain tend to have better survival rates. Additionally, the mycobacterial copper transporter protein B (MctB) was identified as a copper efflux transporter localized on the outer membrane of *Mycobacterium* [32]. Loss of MctB in *M. tuberculosis* drastically reduces the bacterial burden in lungs and lymph nodes. These data suggest that the *Mycobacteria* copper efflux system plays a critical role in virulence.

### 3.2. Bacterial Metallothioneins

Metallothioneins (MTs) are small cysteine-rich peptides that play a critical role in counteracting metal toxicity. They are highly conserved from bacteria to mammals, yet are well-characterized in eukaryotes. Bacterial MT was first identified in *Synechococcus* cyanobacteria using gel permeation and reverse-phase chromatography [33]. The *Synechococcus* MT demonstrated a low-sequence homologous similarity to those from mammals and fungi. However, the MT Cu^+^ coordination motif (Cys-X-Cys) was identified.

The MT of *mycobacterium* (MymT) was identified by screening a genomic library [34]. Similar to that of *Synechococcus*, MymT is a 4.9-kDa protein and contains the Cys-X-Cys motif, indicating a potential Cu^+^ binding function. The preferential binding capacity for MymT is 4–6 Cu^+^ ions. In agreement with this argument, deleting MymT causes hypersensitivity to exogenous copper, resembling the high copper sensitivity phenotype in *Saccharomyces cerevisiae cup1Δ*. However, *mycobacteria* deficient in MymT do not demonstrate attenuation in bacterial pathogenicity in a murine model. Therefore, MTs from pathogenic bacteria serve minor functions in virulence, different from those of fungi that are critical fungal virulence modulators.

### 3.3. Regulation of Pathogenic Bacterial Copper Regulons

The regulation of bacterial copper regulons has differentially evolved between prokaryotic and eukaryotic cells. Eukaryotic cells often employ one of two distinct transcription factors, controlling expression for copper deficiency or copper overload, as appropriate. Examples are Mac1 and Ace1 from *S. cerevisiae* [35,36]. Mac1 responds to low copper levels, and Ace1 responds to high levels. Because an influx system is lacking in pathogenic bacterial cells, no transcriptional responding factors have been identified. The regulation of bacterial regulons discussed in this review is primarily via transcription factors required for copper detoxification. Generally, *Salmonella* and *Mycobacteria* utilize two independent copper-responsive regulon repression mechanisms to precisely regulate the expression of copper efflux and *MT* genes.

In *Salmonella*, the CueR and GolS systems are employed (Figure 1). Both actively bind to the promoter sequences of copper regulons and repress expression. Upon the elevation of intracellular copper, the copper-bound forms of CueR and GolS undergo protein conformational changes and subsequently dissociate from their binding sites, activating the expression of target genes [37]. CueR regulates genes including *cueR*, *copA*, *cueP* (a periplasmic copper-binding protein), and *cuiD* (a periplasmic multicopper oxidase), and GolS controls a gene cluster referred as to *golTBS* [38]. However, regulating the expression of some copper regulons is not only CueR-dependent. One study has demonstrated that the regulation of *cueP* requires an additional system, the CpxR/CpxA two-component system, wherein the activated CueR activates transcription, and CpxR responds by recruiting RNA polymerase [39]. Further investigation showed that regulation of CueR is not only responsive to copper, but also to gold and silver. Moreover, the GolS system has a greater preference for activation in the presence of gold than silver or copper [40]. The GolS metal-binding loop motif (residues 113 to 118) provides a critical function in metal selectivity.

In *Mycobacteria*, the copper efflux system is regulated via CsoR and RicR factors [20] (Figure 1). Crystal structure analysis demonstrates that the copper-bound CsoR protein forms a homodimer structure, and the CsoR apo form shows DNA association activity [41]. CsoR systematically represses the expression of itself and the copper P-type ATPase pump, *ctpV* [42]. Disruption of *csoR* significantly enhances cell growth under toxic copper levels and induces bacterial burdens in the lung, especially at the early stage of infection, because of the induction of the CtpV efflux pump. In addition to copper regulation, the RNA-sequencing analysis in a *csoRΔ* mutant revealed that CsoR also regulates the expression of genes involved in hypoxia and nitric oxide responses [43]. Moreover, the deletion of CsoR shows positive feedback for RicR repression.

Similar to the CsoR system, the RicR protein binds to copper, dampening the repressions of gene expressions of important copper regulons such as *ricR*, *mymT*, *ipqS*, *socAB*, *mmcO*, and Rv2963 [44,45]. Knocking out the *MT* gene in *mycobacteria* does not influence bacterial colonization in the host. In fact, individual RicR-regulated copper regulons demonstrated no obvious copper resistance or virulence phenotypes. For example, an overexpression or deletion of *mmcO* does not confer bacterial burdens in animals. However, a loss of the copper binding mutant of RicR demonstrated copper sensitivity and poor survival in mouse lung tissue, extensively elucidating the critical function of copper regulons during *mycobacteria* invasion.

## 4. Copper Homeostasis in Fungi

Fungal pathogens are major common threads across human communities and are causes of disease and death in humans, animals, and global food crops [46,47,48,49]. Collectively, 1.2 billion people are estimated to be infected with fungal pathogens, and these infections result in 1.6 million deaths annually [46,47]. *Cryptococcus* and *Candida* spp. are important infectious fungal pathogens in mammals. *Cryptococcus neoformans* is an airborne human pathogen widely distributed globally, and it is a causative agent of lung infections [50,51]. As a pulmonary infection progresses, *C. neoformans* can disseminate into the brain, causing lethal meningitis in both healthy and immunodeficient individuals [52,53]. *Candida albicans* is an infectious agent in blood, skin, oral, and gastrointestinal infections [54,55]. Accumulated evidence strongly suggests that fungal copper regulons are critical for fungal pathogenicity during systemic infection. Disrupting critical copper regulons often results in drastic attenuation of fungal fitness in tissues.

### 4.1. Fungal Copper Acquisition Machineries

In contrast to bacterial cuproproteins, the predominant copper-dependent enzymes are localized intracellularly in eukaryotic organisms [4]. The fungal cells have evolved and possess precise and highly efficient copper acquisition, delivery, and storage machineries. Studies of the copper acquisition machinery in the model organism, *S. cerevisiae*, reveal that the Ctr family of transporters includes three copper transporter members: Ctr1, Ctr2, and Ctr3 [56,57,58,59]. While Ctr1 and Ctr3 are functionally redundant high-affinity copper transporters, Ctr2 is defined as a low-affinity copper transporter [4,56,57,58,59,60]. All three are structurally similar and include copper-binding motifs (the N-terminal Met-rich domain), the MX_3_M motif in the second transmembrane region, a C-terminal cysteine-histidine signature, and the ability to form a homotrimer membrane-bound complex [60,61,62]. Ctr1 and Ctr3 are localized on the plasma membrane, pumping copper intracellularly, whereas Ctr2 is evidently localized on the vacuolar membrane, mobilizing vacuolar-stored copper into the cytosol compartment [56,58,61]. The activities of these transporters rely on the presence of the Cu^2+^ metalloreductases Fre1 and Fre2, indicating the specificity of their copper-binding motifs in selecting the reduced copper ion (Cu^+^) [63]. Because copper has potent redox properties, an overload of Cu^+^ ions is highly toxic to the cell; therefore, mobilization of intracellular copper must be tightly regulated. Mobilization of intracellular copper occurs via two copper chaperones, Atx1 and Ccs, which deliver copper to the ATPase Cu^+^ pump (Ccc2) and superoxide dismutase 1 (Sod1), respectively [64,65,66,67,68,69,70,71].

Interestingly, while the transcriptional regulation of copper homeostasis is partially copper-level-dependent (only the expressions of *CTR1* and *CTR3* respond to copper starvation), the expressions of *CCC2*, *CTR2*, and *ATX1* are iron-status-modulated [36,63,72]. The master regulator for copper regulons in *S. cerevisiae* is mediated via transcription factor Mac1 by binding to the copper-responsive elements in the promoters of *CTR1*, *CTR3*, and *FRE1* [63,73].

**Figure 2:**
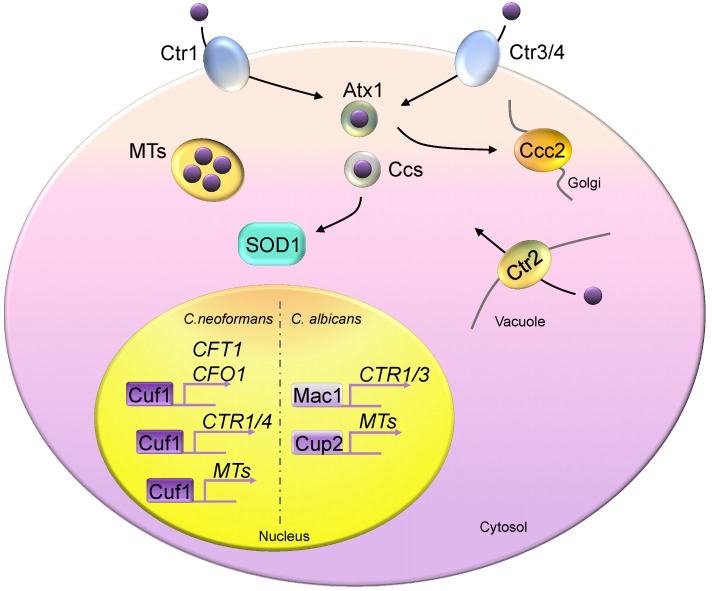
Copper homeostasis in fungi. Fungal cells generally encode two high-affinity copper importers, Ctr1 and Ctr3 (Ctr4), except in *Candida albicans*, which possesses only the Ctr1 homolog. Atx1 and Ccs are two copper chaperones, delivering copper to Ccc2 and Sod1, respectively. Metallothioneins (MTs) are copper detoxification proteins that bind excess copper. Ctr2 is a vacuolar copper transporter, pumping copper from the vacuolar space into the cytosol compartment. Mac1 controls the copper depletion responses of *Saccharomyces cerevisiae* and *C. albicans* by activating the expression of copper transporters. *Saccharomyces cerevisiae* Ace1 responds to copper toxicity and induces MT expression. *C. neoformans* Cuf1 controls responses in both copper-rich and copper-poor environments. Cuf1 constitutively binds to the promoter sequences of *CFT1* and *CFO1*.

The study of copper homeostasis in *S. cerevisiae* provides a fundamental basis for understanding the function of copper in human fungal pathogens. Generally, the Ctr family is highly conserved in pathogenic fungi [19,74,75,76,77,78,79]. A phylogenetic analysis using 300 fungal species demonstrated that fungal genomes encode at least one Cu^+^ importer (either Ctr1 or Ctr3) [76]. The genome of the meningitis fungal pathogen *C. neoformans* has been shown to encode two functionally redundant copper importers, referred as to Ctr1 and Ctr4 (Figure 2). The expression of either *CTR1* or *CTR4* in *C. neoformans* rescues the respiratory defects in *S. cerevisiae ctr1Δ ctr3Δ*, strongly suggesting that both these copper transporters act as copper importers [19,61,62]. Similar to *S. cerevisiae*, gene expressions of *CTR1* and *CTR4* respond to a copper-deficient environment (in the presence of a copper chelator), and this expression is activated via the transcription factor Cuf1, a homolog of Mac1 from *S. cerevisiae*. Disruption of either *CTR1* and *CTR4* or *CUF1* will abolish cell growth under low copper levels [75,76]. In *C. neoformans*, the Atx1 and Ccc2 homologs were first identified using the *Agrobacterium*-mediated insertional mutation library, which was designed for screening important players in melanin formation. The insertion mutants of *ATX1* and *CCC2* demonstrated impaired cell growth in low-iron conditions. Moreover, *ccc2Δ* mutants impaired fungal bisexual mating [80].

The genome of *C. albicans* encodes only *CTR1*; evidently, *CTR3* was lost during evolution [76]. This phenomenon can be examined across almost all *Candida* species. A likely explanation is that diploid *Candida* species must reduce the number of copper transporter genes so that intracellular copper overload can be prevented. The experimental evidence that supports this argument is that the heterozygous and homozygous mutants of *CTR1* induce hyphal growth in a gene-dosage manner [79]. In addition to the Ctr family, an alternative copper uptake machinery has been suggested in *C. albicans* via two previously defined cell surface adhesion proteins, Als1 and Als3. Zheng et al. demonstrated that the *als1ΔΔ* and *als3ΔΔ* mutants exhibit an enhanced biofilm formation and cell survival rate on the copper surface, indicating that they become copper resistant [81]. In turn, intracellular copper content was significantly reduced in these mutants. Furthermore, the expression of the *C. albicans* detoxification gene, *CRP1*, is induced in *als1ΔΔ* and *als3ΔΔ* mutants, yet the regulation mechanism remains unclear [81].

### 4.2. Fungal Copper Detoxification Machineries

Much attention has been drawn toward the detoxification mechanisms in pathogens, given the suggestion by mounting evidence that copper presents an anti-fungal bombardment during infection [19,75,82]. Despite the critical biological function of copper, an environment of excess copper is highly toxic to fungal cells. Copper overload is associated with the generation of free radical stress via the Fenton reaction, and it importantly destroys the Fe-S cluster [5,82,83,84]. Resembling bacteria, fungal cells evolved to provide efficient yet species-unique mechanisms for protecting against copper toxicity. The highly conserved detoxification machinery from prokaryotes to eukaryotes is the neutralization of toxic copper by MTs [85,86]. Though they are highly conserved in their cysteine-rich property, this is not necessarily so for their protein sequences. The gene expression of fungal MTs is positively regulated by the exogenous copper concentration [87]. In *S. cerevisiae* and *C. neoformans*, MTs are the primary and predominant copper detoxification approach, whereas in *C. neoformans*, MTs are atypical proteins demonstrating an extraordinary copper-scavenger ability. *Cryptococcus neoformans* MTs can coordinate up to 24 Cu^+^ ions via cysteine residues [75,76,88].

Although the MTs of *C. albicans* are smaller and weaker copper-scavenger proteins (Cup1 and Crd2) compared to those of *C. neoformans* [76,89], this fungus has evolved to use additional machineries for detoxifying excess copper. Resembling the copper exportation mechanism in bacteria, *C. albicans* utilizes a membrane-bound P-type ATPase (Crp1) for the efflux of intracellular copper [90].

While MTs are considered the first line of defense against copper toxicity, recent work has identified a novel fungal-conserved copper detoxification machinery that provides Fe-S cluster protein biogenesis [82]. In response to an elevation of intracellular copper levels, *C. neoformans* induces the expression of a gene encoding a mitochondrial ABC transporter (Atm1), and it induces gene expression in a Cuf1-dependent manner. Atm1 is an Fe-S cluster pump, delivering Fe-S from mitochondria to the cytosol compartment. In turn, the disruption of *ATM1* gene reduces cell resistance to high copper levels. Protection from copper toxicity by Atx1 occurs in spite of the overexpression of MTs, suggesting that fungal cells employ multiple independent processes to counteract a copper overload [82].

### 4.3. Regulation of Fungal Copper Regulons

The regulation of copper regulons in fungi is achieved primarily through transcriptional modulation of gene expression via copper-responsive transcription factors. Transcription factors such as Mac1, Ace1, and Cuf1 contain cysteine-rich domains and direct Cu^+^ binding motifs [35,36,76,91,92,93]. The binding of Cu^+^ to a transcription factor triggers structural alterations in the protein, thus leading to either activation or repression of transcription factor activities. *Saccharomyces cerevisiae* uses two functionally reciprocal transcription factors, Mac1 and Ace1, for modulating copper responses. Mac1 is a copper-depletion responsive transcription factor, activating gene expression and encoding copper acquisition proteins such as *CTR1*, *CTR3*, and *FRE1* [36]. It coordinates up to 8 Cu^+^ ions via two distinct cysteine-rich domains. The formation of a copper cluster in Mac1 results in protein inactivation and degradation at high copper levels, rapidly shutting down the expression of copper transporters [11,91]. *Saccharomyces cerevisiae* and *C. albicans* cells without Mac1 demonstrate growth impairment in a copper-limited environment [94]. Ace1 was identified as a transcription activator for the expression of copper detoxification genes. In response to high copper levels, Ace1 binds to the promoters of target genes via specific metal-responsive elements, with the consensus sequence of 5′-HTHNNGCTGD-3′ (D = A, G, or T; H = A, C, or T), which can be found in the promoter regions of MT genes and *SOD1* [95,96,97].

In *C. neoformans*, Cuf1 was first identified as a Mac1-like transcription factor, participating in the activation of Ctr4, a homolog of Ctr3 from *S. cerevisiae* [78] (Figure 2). However, a later study reported a copper-sensitive phenotype in a *cuf1Δ* mutant and demonstrated that Cuf1 is required for activating gene expression for Ctr1, Ctr4, and MTs [75,76,93]. Cuf1 is regulated on these target genes via a direct protein-DNA interaction. These data extensively suggest the dual function of Cuf1 in balancing copper homeostasis in *C. neoformans* whether under copper repletion or copper depletion conditions. A genome-wide analysis using RNA-seq and ChIP-seq data for Cuf1 reveals its intriguingly complex regulation pattern [93]. The bindings of Cuf1 to a promoter of copper-responsive genes were constitutive, with minor re-localizations of target sequence bindings upon copper treatments. For example, Cuf1 binds to the promoters of Ctr1 and Ctr4 under a copper-limited environment, and it coordinates with the promoters of *MT1* and *MT2* in more copper-rich environments, constitutively binding to those of *CFT1* (*Cryptococcus* Fe transporter 1) and *CFO1* (*Cryptococcus ferroxidase 1*). Binding motif computational analysis demonstrated that the consensus sequence in copper-poor conditions were similar between Cuf1 and Mac1 from *S. cerevisiae* or *C. albicans*. However, no significantly enriched Cuf1 binding motif was identified for regulating high-copper environments.

Fungal cells also employ alternative regulation machineries including protein modification and degradation and G-protein-coupled receptor signaling for tightly regulating copper homeostasis. A recent study of copper transporters in *C. neoformans* revealed two protein species for Ctr4, and further analysis demonstrated that glycosylation was modified for Ctr4 [19]. While this glycosylation is not involved in protein trafficking, a non-glycosylated protein attenuates in copper uptake, suggesting that the presence of sugar somehow contributes to copper binding at the extracellular copper-binding domain of Ctr4. Additionally, the glycosylated Ctr4 tends to be more stable in the presence of exogenous copper. Even though the degradation processes for copper transporters remain unknown, Ctr1 and Ctr4 are highly unstable in copper-rich environments [19,98]. *Candida albicans* Gpa2, a G-protein α subunit, was identified upstream in the cyclic AMP protein kinase A pathway and was found to govern copper detoxification regulation, probably by dampening the expression of copper acquisition while activating it for MTs. The copper resistance phenotype can be reversibly abolished by artificially adding cAMP [98].

### 4.4. Fungal Copper-Dependent Virulence Factors

Fungal fitness at the pathogen-host axis is defined as successful colonization, proliferation, and invasion during systemic infection [52,53,99]. During the life cycles of fungi in the host, virulence factors play vital roles in counteracting the harsh and hostile environment. Many of these factors are cuproproteins, and copper serves as the critical determinant for their functions. Identified virulence-associated cuproproteins include superoxide dismutase (SOD), laccase, copper-responsive transcriptional regulators, copper detoxification mechanisms, and copper chaperones [75,78,80,82,100,101,102].

Generating reactive oxygen species (ROSs) in a hostile environment, particularly within the phagosomal compartment, is the first line of immune defense against invading microbes [5]. The ability of fungal cells to neutralize ROS toxicity is an essential function for fungal fitness in host immune cells. Copper-bound SODs are potent ROS detoxification agents that use copper as a redox center, converting the superoxide anion to hydrogen peroxide, which eventually is catalyzed to water and oxygen. Of many types of SODs, the Cu, Zn Sod1 is the predominant ROS scavenger in fungi. *C. neoformans*, lacking Sod1, dramatically attenuates fungal pathogenicity in a murine model. Cell-surfaced *C. albicans* SODs include 2 Cu, Zn SODs (Sod4 and Sod6) and the copper-only Sod5 [103,104].

Many organisms can generate the pigment melanin. Fungal melanin demonstrates its critical roles in ROS detoxification and UV radiation absorption [99]. Lately, *C. neoformans* melanin has been demonstrated to govern iron acquisition, and its formation process is catalyzed by copper-bound laccase using L-DOPA as a melanin precursor [105]. The loading of copper to *C. neoformans* laccase is mediated by the copper chaperone Atx1 and the P-type ATPase Ccc2. Insertional mutagenesis of *ATX1* and *CCC2* results in pigment formation defects, resembling those from a *lac1Δ* [80].

The expression of many copper-dependent virulence factors is transcriptionally regulated. The ChIP-seq analysis in *C. neoformans* from Cuf1 reveals that Cuf1 directly binds to the promoter sequences of Lac1, Sod1, Atm1, and other important copper-related genes [93,94]. In other pathogenic fungi, copper-responsive transcriptional regulation is also associated with the production of virulence factors. *Candida albicans* Mac1 is a negative regulator for hyphal formation, probably through the inhibition of iron acquisition machineries, a known hyphal formation inducer [94].

### 4.5. Copper at the Fungal Pathogen-Host Axis

The host has developed a sophisticated mechanism for metal manipulation to battle invading microbes. Research has demonstrated the sequestration of iron and zinc at the host-pathogen axis via activation of the iron pump Nramp1 on the phagosomal compartment and secretion of a chelation peptide (e.g., calprotectin) to lower metal levels to microbes [106,107]. In contrast to iron and zinc, copper is thought to be used as an immune bombardment [10]. Evidence that supports this hypothesis is the accumulation of phagosomal copper content in mycobacteria-infected macrophages. Further analysis revealed that the activation of macrophage-like cells by interferon-gamma or bacterial cell wall components (lipopolysaccharides) triggers protein elevation for Ctr1 and ATP7A, of which the latter relocalized to the membrane of a phagolysosome, further proving that copper is used as an anti-microbial agent [17].

While these experiments were performed in vivo, recent studies employing *C. neoformans* copper-responsive reporter constructs, live animal imaging, and two-photon microscopy demonstrated that the MT gene promoter is specifically activated, whereas that of the copper transporter is repressed [75]. These data suggest that fungal cells experience copper elevation during pulmonary infection. In turn, the lack of MT genes in fungal cells shows attenuated fitness in the lung. Intriguingly, copper transporter gene knockout strains demonstrated interesting fungal burden profiles, showing increased cell colonization in the lung but significantly reduced fungal fitness in the brain [19]. These phenomena suggest that the selection of an anti-fungal copper machinery for activation depends on infection niches.

In fact, the mobilization of copper during a fungal infection is a highly dynamic process. Despite the generally accepted of hypothesis, that Cu acts as a bombardment, several important studies highlighted the critical role of host Cu starvation in limiting pathogen proliferation in tissue [108]. Examples include the argument that host brain tissue limited Cu content during the formation of cryptococcal meningitis. A further study, using *C. albicans* kidney and renal infection models, Mackie et al. showed increased copper levels in tissue at an early stage of infection, leading to an increased expression of a *C. albicans* gene-encoding copper efflux pump but repression of gene expression of *CTR1* [18]. However, a reciprocal regulation of copper homeostasis was observed in *C. albicans* cells at a late stage of infection. Copper depletion in the kidney is most likely the result of secretion of a transition metal binding protein, calprotectin. While calprotectin is a zinc sequestration molecule, its copper binding activity has been identified [109]. Kidney-invading *C. albicans* cells induce copper-starvation-required genes such as *SOD1* and *SOD3* in response to calprotectin. However, copper chelation by calprotectin is transient, progressing within 72 h.

## 5. Conclusions

It is fascinating how life precisely handles toxic intracellular Cu^+^ and how the host uses it as an efficient anti-microbial agent. Studying and comparing copper regulons among bacterial and fungal species provides valuable information about how copper homeostasis evolves in nature. A clear boundary exists in copper homeostasis, the copper importing system, between prokaryotes and eukaryotes. The system is obviously lacking in bacteria. How and where do eukaryotic cells gain the genes to encode copper importers and low-copper responsive regulation, as in the Ctr family and Mac1? Why do most pathogenic fungi (except *C. albicans*) lose the copper efflux system? In addition, bacterial species generally employ two independent regulation machineries to regulate copper regulons, such as CsoR and RicR from *mycobacteria*, whereas fungal cells use a solo regulation system, such as Mac1 from *S. cerevisiae* and Cuf1 of *C. neoformans*. Monitoring copper homeostasis at the host-pathogen axis is critical; however, the study of microbial copper regulons has come to the center stage with little understanding of the host. Hypotheses about the role of host copper in defending microbes remain controversial. However, it appears that mounting evidence points to copper bombardment rather than copper sequestration as the technique used by host cells. To extensively dissect the function of Cu in defending pathogen infections and to elucidate the impacts between Cu bombardment and Cu starvation, further studies using copper regulons from genetically modified animals are crucial.
